# Supplementary material for: Identification and characterization of intermediate states in mammalian neural crest cell epithelial to mesenchymal transition and delamination
Source: bioRxiv. 2024 Feb 15:2023.10.26.564204. Originally published 2023 Oct 26. Preprint. [Version 2] doi: 10.1101/2023.10.26.564204 (PMC10634855; doi:10.1101/2023.10.26.564204)
Supplement: Supplement 1 [file NIHPP2023.10.26.564204v2-supplement-1.pdf]

bioRxiv preprint doi: https://doi.org/10.1101/2023.10.26.564204; this version posted February 15, 2024. The copyright holder for this preprint (which was not certified by peer review) is the author/funder, who has granted bioRxiv a license to display the preprint in perpetuity. It is made available under aCC-BY 4.0 International license.

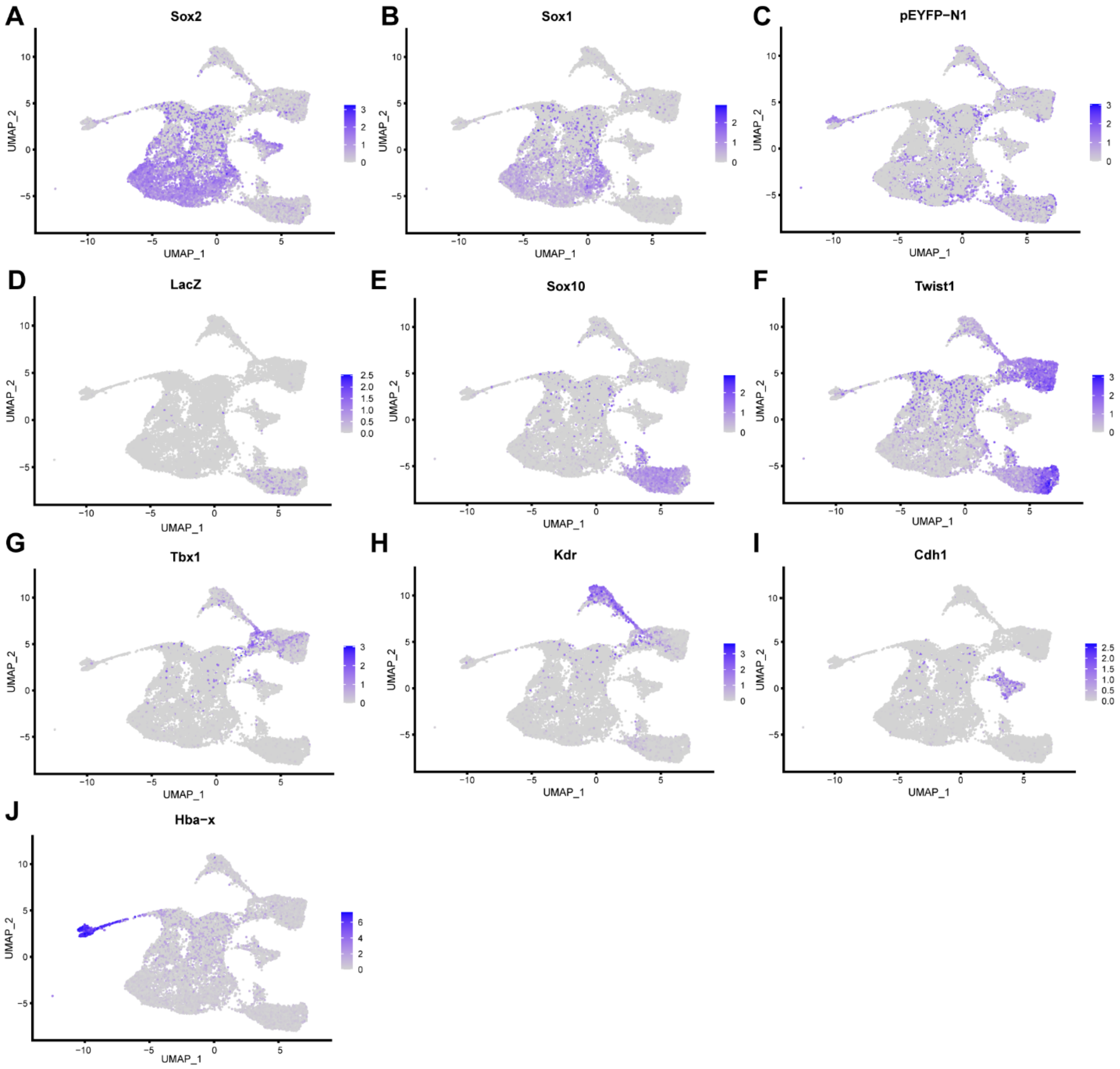

Figure 1-figure supplement 1. Expression of tissue specific marker genes that identify 6 major cell type clusters in early E8.5 mouse embryonic cranial tissues. FeaturePlots that show the expression of (A) *Sox2*, neuroectoderm marker; (B) *Sox1*, neuroectoderm marker; (C) *eYFP*, premigratory and migratory NCC marker; (D) *LacZ*, migratory NCC marker; (E) *Sox10*, migratory NCC marker; (F) *Twist1*, NCC and mesoderm marker; (G) *Tbx1*, mesoderm marker; (H) *Kdr*, endothelial cell marker; (I) *Cdh1*, non-neural ectoderm marker; (J) *Hba-x*, embryonic blood cell marker.

bioRxiv preprint doi: https://doi.org/10.1101/2023.10.26.564204; this version posted February 15, 2024. The copyright holder for this preprint (which was not certified by peer review) is the author/funder, who has granted bioRxiv a license to display the preprint in perpetuity. It is made available under aCC-BY 4.0 International license.

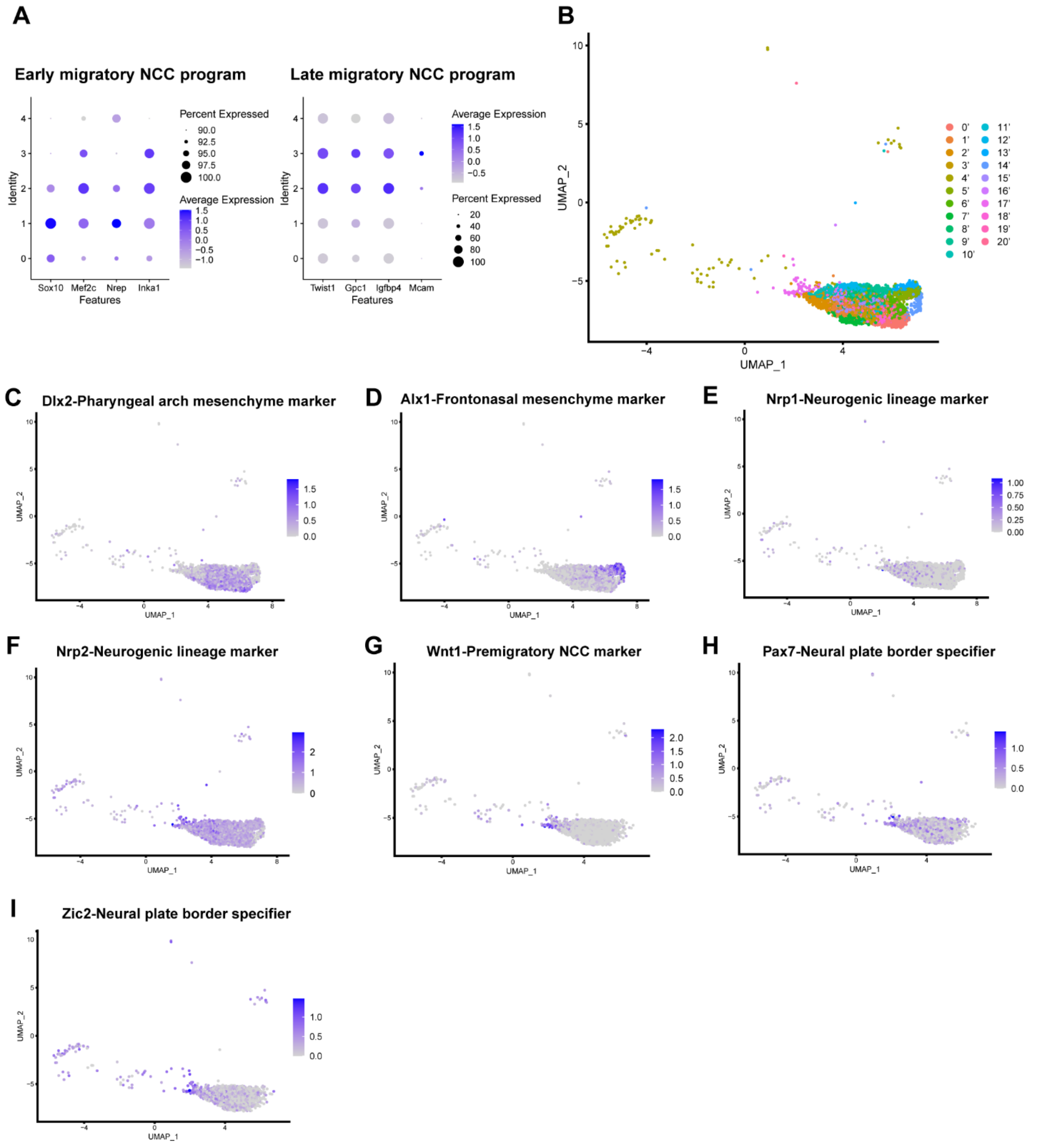

Figure 2-figure supplement 1. Additional gene expressions that support the identity of cranial NCC subclusters at 0.26 and 2.0 resolutions. (A) Expression of NCC development program genes (Soldatov et al., 2019) confirms NCC subcluster 0 and 1 as early migratory NCC and subcluster 2 and 3 as late migratory NCC. Dotplots showing the expression of early and late NCC program genes in 5 NCC subclusters at resolution 0.26. Subcluster 0-3 all express early migratory NCC program genes, but only subcluster 2 and 3 express a significant level of late migratory NCC program genes. (B) UMAP and re-clustering of the cranial NCC cluster into 21 smaller

bioRxiv preprint doi: https://doi.org/10.1101/2023.10.26.564204; this version posted February 15, 2024. The copyright holder for this preprint (which was not certified by peer review) is the author/funder, who has granted bioRxiv a license to display the preprint in perpetuity. It is made available under aCC-BY 4.0 International license.

subclusters at a resolution of 2.0. (C-F) FeaturePlots show the expression of mesenchyme and neurogenic lineage markers in cranial NCC. Subcluster 2 displays a high expression of pharyngeal arch mesenchyme marker *Dlx2* (C). The frontonasal mesenchyme marker *Alx1* is specifically expressed by subcluster 3 (D). Neurogenic lineage markers *Nrp1* and *Nrp2* are expressed in late migratory NCC (E and F). (G-I) FeaturePlots showing expression of the premigratory NCC marker *Wnt1* (G) and neural plate border specifiers *Pax7 and Zic2* (H and I) in early migratory NCC. Subcluster 2' and 10' cells express reduced levels of *Wnt1*, *Zic2* and *Pax7* than subcluster 17' cells.

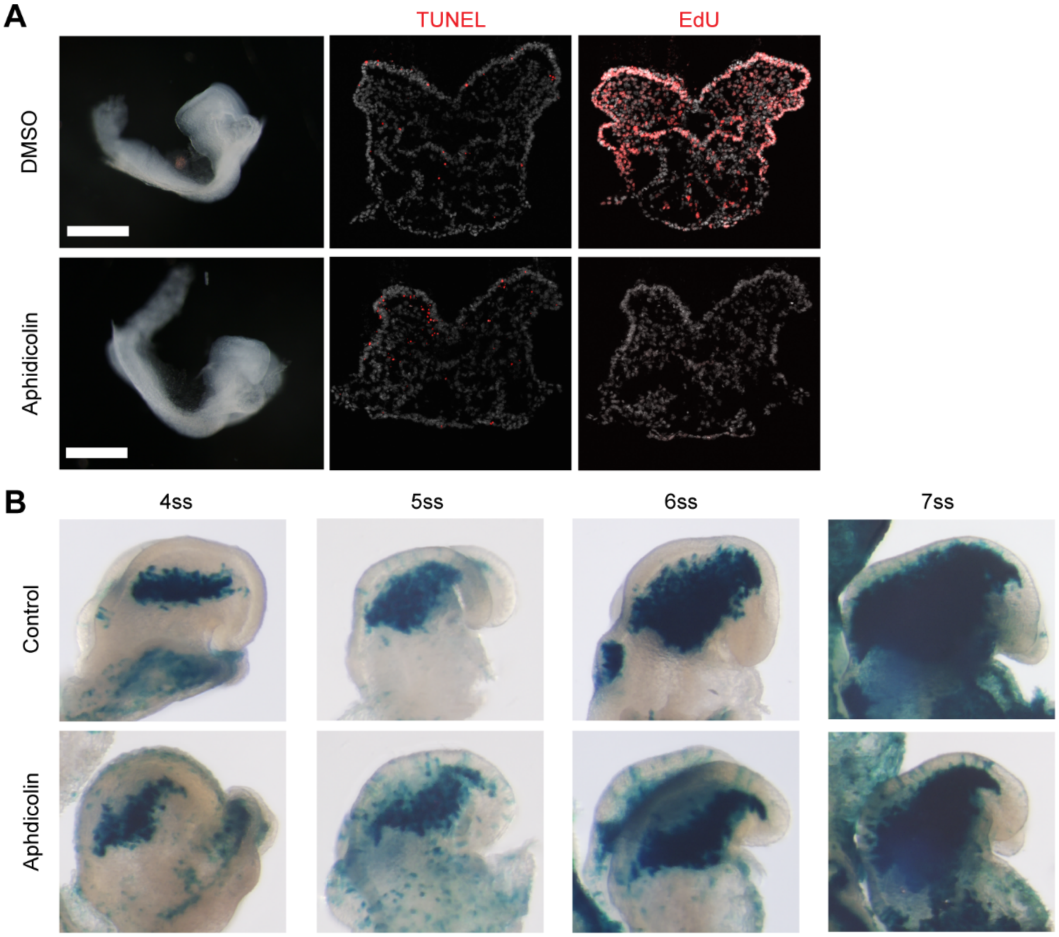

Figure 4-figure supplement 1. Aphidicolin treatment on *Mef2c-F10N-LacZ* embryos show consistent results as CD1 embryos. (A) Aphidicolin treatment in early E8.5 CD1 embryos for 12-13 hours prevented cells from entering S phase cell cycle and induced minimal cell death. Aphidicolin treated embryos exhibit a lack of EdU incorporation and a similar level of TUNEL signal compared to the control. (B) *Mef2c-F10N-LacZ* embryos treated with Aphidicolin for 12-13 hours exhibit reduced migratory NCC as evidenced by β-galactosidase staining.

bioRxiv preprint doi: https://doi.org/10.1101/2023.10.26.564204; this version posted February 15, 2024. The copyright holder for this preprint (which was not certified by peer review) is the author/funder, who has granted bioRxiv a license to display the preprint in perpetuity. It is made available under aCC-BY 4.0 International license.

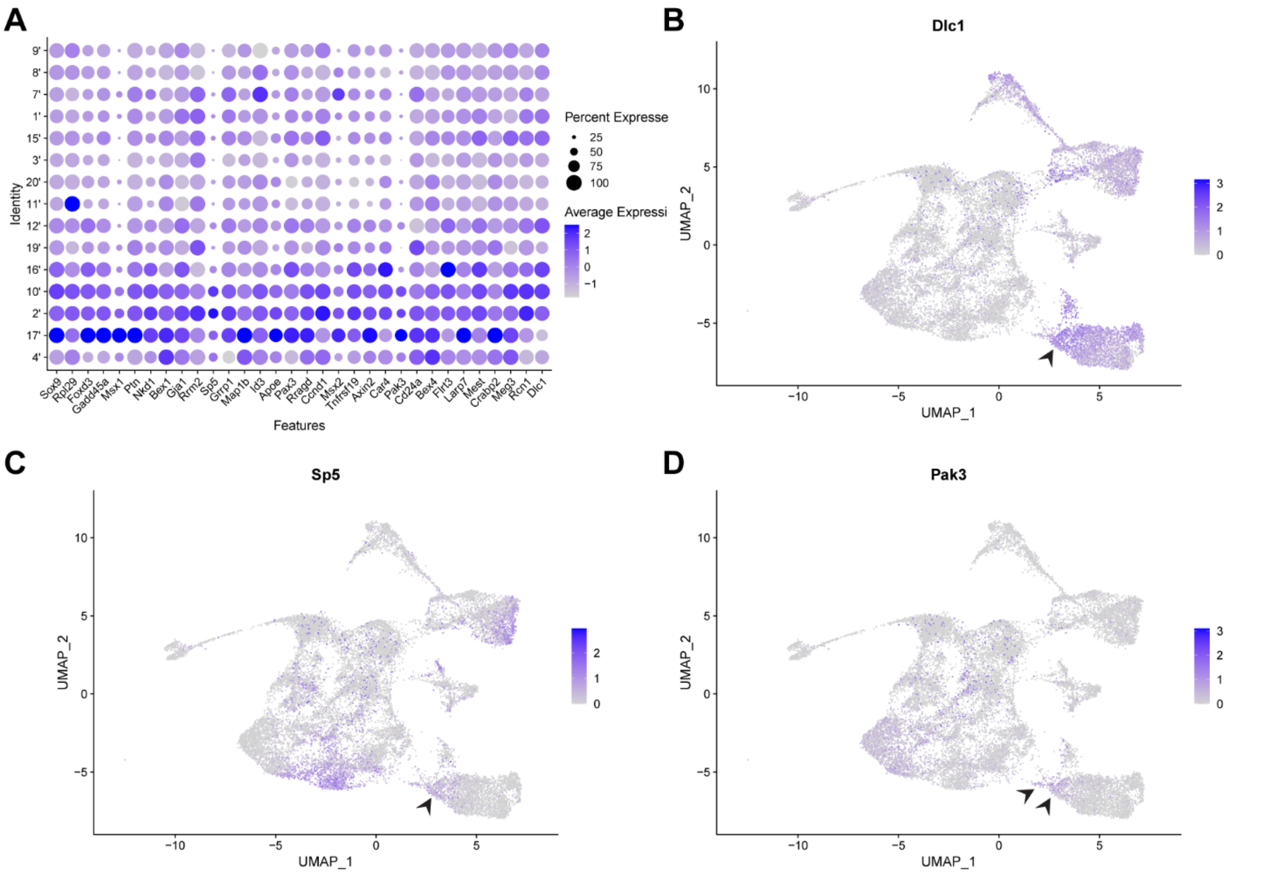

Figure 5-figure supplement 1. Expression of EMT intermediate NCC markers in scRNA-seq data. (A) Dotplot showing the expression of all EMT intermediate NCC markers in early migratory NCC subclusters (resolution 2.0). (B-D) FeaturePlots showing the expression of *Dlc1*, *Sp5* and *Pak3* in all early E8.5 cranial mouse embryo tissues. The expression of *Dlc1*, *Sp5* and *Pak3* is largely restricted to NCC, neuroectoderm and mesoderm cells. Arrowheads indicate *Dlc1*, *Sp5* and *Pak3* expression specifically in premigratory and intermediate NCC.

bioRxiv preprint doi: https://doi.org/10.1101/2023.10.26.564204; this version posted February 15, 2024. The copyright holder for this preprint (which was not certified by peer review) is the author/funder, who has granted bioRxiv a license to display the preprint in perpetuity. It is made available under aCC-BY 4.0 International license.

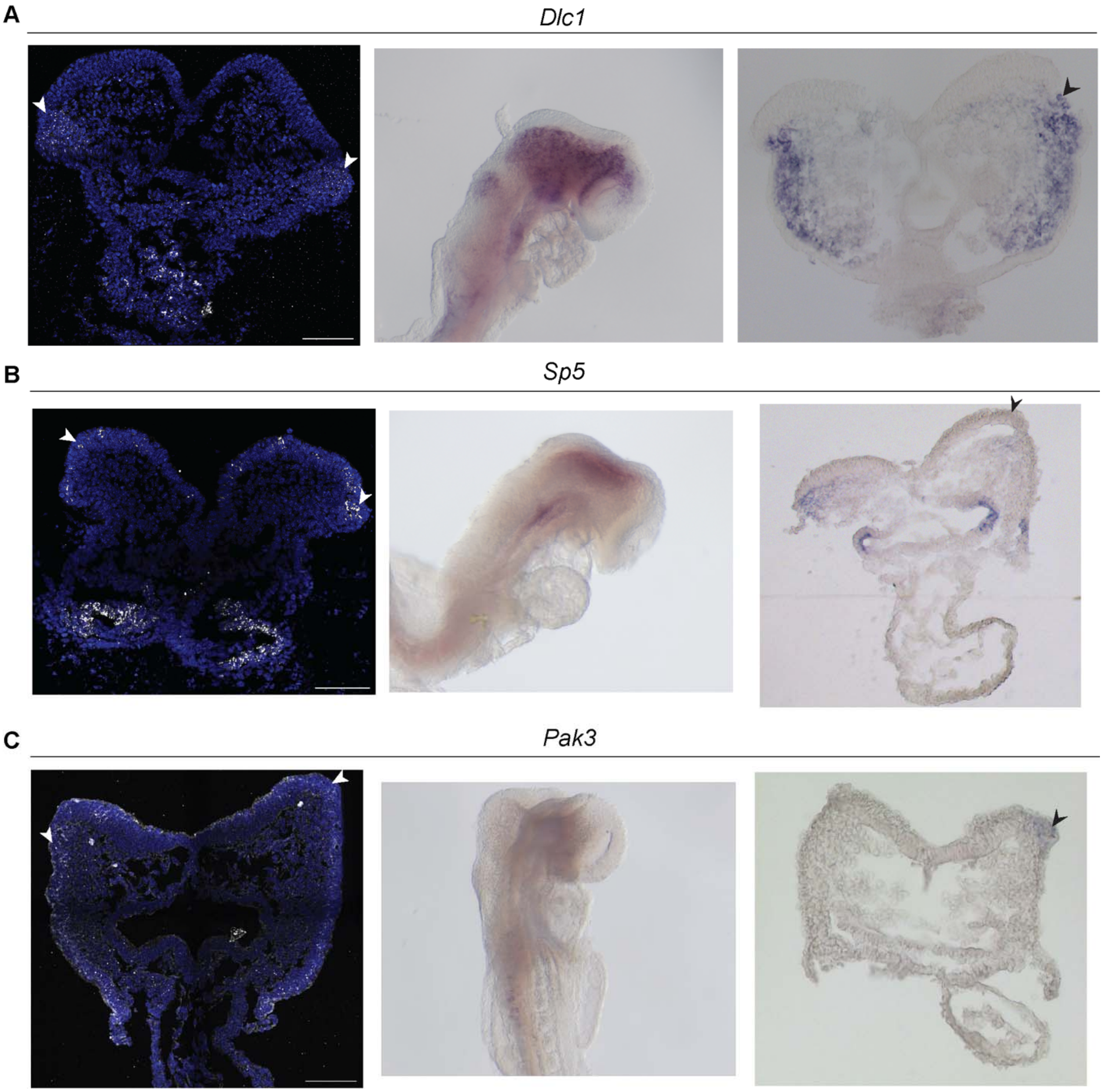

Figure 5-figure supplement 2. Expression of intermediate NCC markers *Dlc1*, *Sp5* and *Pak3* by SABER-FISH and traditional in situ hybridization in E8.5 mouse embryos and cranial sections. Arrowheads indicate positive signals in the dorsolateral neuroepithelium. (A) *Dlc1* signal was observed in the dorsolateral region of the neuroepithelium as well as in cells just outside of the neuroepithelium in the underlying mesenchyme. (B) *Sp5* expression is localized within the neuroepithelium and underlying dorsal mesenchyme. (C) *Pak3* expression is observed is ectodermal tissues including the neuroectoderm or neuroepithelium.

bioRxiv preprint doi: https://doi.org/10.1101/2023.10.26.564204; this version posted February 15, 2024. The copyright holder for this preprint (which was not certified by peer review) is the author/funder, who has granted bioRxiv a license to display the preprint in perpetuity. It is made available under aCC-BY 4.0 International license.

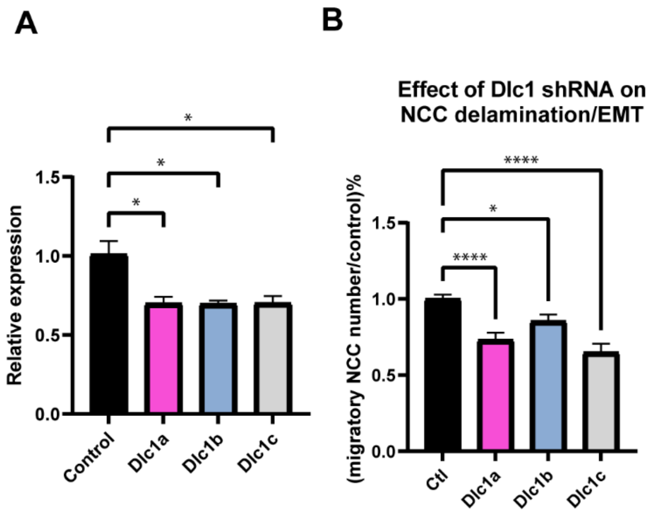

Figure 6-figure supplement 1. *Dlc1* plays a regulatory role in mouse cranial NCC EMT and delamination. (A) *Dlc1a*, *Dlc1b* and *Dlc1c* shRNA-based lentiviruses consistently achieved 30% reduction of *Dlc1* expression based on qRT-PCR analysis. *p<0.05. (B) Embryos injected with *Dlc1a*, *Dlc1b* and *Dlc1c* shRNA-based lentiviruses consistently showed significantly fewer migratory NCC compared to the control. The number of Sox10+ migratory NCC was quantified in control (n=4), *Dlc1a* (n=4), *Dlc1b* (n=4) and *Dlc1c* (n=4) embryos. All datapoints in *Dlc1a*, *Dlc1b* and *Dlc1c* samples were normalized to the control samples. *p<0.05. ****p<0.0001.

Supplementary Table 1. NCC development related genes.

Gene	Expression pattern in relation to NCC development	References
Sox2	Neural epithelium (reduced in neural plate border)	Hafemeister & Satija, 2019; Lee et al., 2013; Wood & Episkopou, 1999
Sox1	Neural epithelium (reduced in neural plate border)	Hafemeister & Satija, 2019; Lee et al., 2013; Wood & Episkopou, 1999
Zic1/2	Neuroepithelium	Sauka-Spengler & Bronner-Fraser, 2008
Wnt1	Neural plate border	Echelard et al., 1994; Parr et al., 1993
Pax7	Neural plate border	Murdoch et al., 2012
GCNF (Nr6a1)	Neural epithelium and early MNCC	
Zeb2	Neural epithelium and early MNCC	Van de Putte et al., 2003
Pax3	Neural plate border and early MNCC	Li et al., 2000
(Wnt1-Cre) EYFP	Neural plate border and early MNCC	Hari et al., 2012
Sox9	Neural plate border and early MNCC	Lee et al., 2013
Foxd3	Neural plate border and early MNCC	Dottori et al., 2001

bioRxiv preprint doi: https://doi.org/10.1101/2023.10.26.564204; this version posted February 15, 2024. The copyright holder for this preprint (which was not certified by peer review) is the author/funder, who has granted bioRxiv a license to display the preprint in perpetuity. It is made available under aCC-BY 4.0 International license.

Snail1	Neural plate border and early MNCC	Cheung et al., 2005
Mef2c-F10N-LacZ	Predominantly MNCC	Aoto et al., 2015
Vimentin	MNCC (marker of mesenchymal cells)	Kobayashi et al., 2020
Sox10	MNCC	Hari et al., 2012
Twist1	MNCC	Soo et al., 2002

Supplementary Table 2. Primers for qRT-PCR

	Forward (5'-3')	Reverse (5'-3')
Dlc1	AGCGGCTGTGAAAGAAA	GCATTACCCTTGGAGAAGA
B2M	CACTGACCGGCCTGTATGC	GGTGGCGTGAGTATACTTGAATTTG
CANX	CCAGACCCTGATGCAGAGAAG	CCTCCCATTCTCCGTCCATA
